# The Regenerative Capacity of the Zebrafish Caudal Fin Is Not Affected by Repeated Amputations

**DOI:** 10.1371/journal.pone.0022820

**Published:** 2011-07-28

**Authors:** Ana Sofia Azevedo, Bartholomäus Grotek, António Jacinto, Gilbert Weidinger, Leonor Saúde

**Affiliations:** 1 Instituto de Medicina Molecular e Instituto de Histologia e Biologia do Desenvolvimento, Faculdade de Medicina da Universidade de Lisboa, Lisboa, Portugal; 2 Instituto Gulbenkian de Ciência, Oeiras, Portugal; 3 Centro de Neurociências e Biologia Celular, Universidade de Coimbra, Coimbra, Portugal; 4 Biotechnology Center and Center for Regenerative Therapies, University of Technology Dresden, Dresden, Germany; Center for Regenerative Therapies Dresden, Germany

## Abstract

**Background:**

The zebrafish has the capacity to regenerate many tissues and organs. The caudal fin is one of the most convenient tissues to approach experimentally due to its accessibility, simple structure and fast regeneration. In this work we investigate how the regenerative capacity is affected by recurrent fin amputations and by experimental manipulations that block regeneration.

**Methodology/Principal Findings:**

We show that consecutive repeated amputations of zebrafish caudal fin do not reduce its regeneration capacity and do not compromise any of the successive regeneration steps: wound healing, blastema formation and regenerative outgrowth. Interfering with Wnt/ß-catenin signalling using heat-shock-mediated overexpression of Dickkopf1 completely blocks fin regeneration. Notably, if these fins were re-amputated at the non-inhibitory temperature, the regenerated caudal fin reached the original length, even after several rounds of consecutive Wnt/ß-catenin signalling inhibition and re-amputation.

**Conclusions/Significance:**

We show that the caudal fin has an almost unlimited capacity to regenerate. Even after inhibition of regeneration caused by the loss of Wnt/ß-catenin signalling, a new amputation resets the regeneration capacity within the caudal fin, suggesting that blastema formation does not depend on a pool of stem/progenitor cells that require Wnt/ß-catenin signalling for their survival.

## Introduction

In contrast to humans, some organisms retain the extraordinary capacity to regenerate throughout adult life. One of such organisms is the zebrafish, a vertebrate that is able to regenerate fins, scales, retina, spinal cord and heart among other internal organs [1].

Due to its accessibility, its fast and robust regeneration and its simple architecture, the zebrafish caudal fin is one of the most powerful models for regenerative studies. The caudal fin is composed of several segmented bony rays and inter-ray mesenchymal tissue, all enclosed by an epidermis. Each bony ray consists of 2 concave hemirays that define an inner space filled with intra-ray mesenchymal cells. Blood vessels and nerve axons are found in both intra- and inter-ray tissues [2]. Bony rays are produced and maintained by osteoblasts (also called scleroblasts), skeletogenic cells that secrete bone matrix [3].

When a caudal fin is amputated, a regenerative program with stereotypic successive steps is activated and it takes approximately 2 weeks to fully regenerate all the tissues and structures that compose a functional fin. Within 1–3 hours-post-amputation (hpa), epithelial cells migrate to cover and close the wound. By 18–24 hpa, an apical epidermal cap (AEC) is formed and a mass of undifferentiated mesenchymal cells called the blastema accumulates underneath the AEC [2]. At 24 hpa the blastema cells segregate into two morphologically indistinct compartments: a slowly proliferating distal blastema and a rapidly proliferating proximal blastema. The distal blastema contributes with daughter cells to the proximal blastema, which is a population of cells that migrate to new positions and differentiate to replace the lost tissues. After 48 hpa the regeneration program is installed and the regenerative outgrowth continues until the original tissue architecture is reconstituted [4].

The capacity to make and organize a blastema is a shared feature of all organisms that are able to efficiently regenerate upon appendage amputation. Although the active cell proliferation of the blastema is required for the progression of regeneration, little is known about the origin and fate of the blastema cells in the fish fin. Regarding the origin of blastema cells, we could consider two hypotheses. One possibility is that stem/progenitor cells become activated upon amputation and migrate distally to form the blastema. While stem cells are the source of regenerating tissues in invertebrates such as planarians and annelids among others [5], little evidence for the contribution of resident stem cells to the formation of the blastema has been obtained in vertebrate appendage regeneration, with the exception of a potential role of muscle satellite cells in salamander limb regeneration [6]. Another possibility that has been proposed to occur in urodele amphibians is that blastema cells originate from a process of dedifferentiation of adult differentiated cells [7]. Lineage tracing analysis using injection of dyes has suggested that muscle fibers disintegrate and that cells containing the dye are found in the forming blastema in regenerating urodele limbs [8], [9]. However, whether muscle-derived cells contribute to the forming regenerate has not been shown. Thus, in vivo evidence for the contribution of mature differentiated cells to appendage regeneration based on molecular markers of the cellular differentiation status and genetic lineage tracing is lacking for the salamander. We have recently used such tools to address the cellular mechanism of bone regeneration in the zebrafish caudal fin [10]. Interestingly, we found that mature osteoblasts dedifferentiate to form part of the appendage blastema. Osteoblast-derived blastema cells remain lineage restricted and give rise only to osteoblasts in the regenerating fin. Thus, strong evidence for mature cells as the source of regenerating vertebrate appendages is starting to accumulate. Other recent studies have shown that other cell lineages also retain their fate when they go through a regenerative process in the zebrafish fin [11] and in the salamander limb [12]. Therefore, transdifferentiation from one lineage into another does not occur during vertebrate appendage regeneration and blastema cells, whether they form by dedifferentiation or from progenitor cells, do not appear to be multipotent.

Regeneration of a complex organ must involve a number of signalling pathways to coordinate blastema formation, cell proliferation, differentiation and patterning events. Although we are beginning to understand the molecular mechanisms of regeneration, it is becoming clear that signalling pathways such as Hedgehog (Hh), Fibroblast growth factor (Fgf) and Wnt among other molecules are activated upon amputation and control different aspects of caudal fin regeneration in zebrafish [1], [13]. Fin regeneration is impaired due to a reduction in cell proliferation when Hh signalling is disrupted by inhibiting its receptor Smoothened using cyclopamine. Conversely, the ectopic overexpression of *sonic hedgehog* (*shh*) leads to excessive bone deposition in regenerating fins, suggesting a role in proliferation and differentiation of bone-secreting cells [14]. The formation of the blastema is impaired in *fgf20a* mutants, when Fgfr1 is pharmacologically inhibited and in a transgenic line expressing a dominant-negative Fgfr1, [15], [16], [17]. The Wnt signalling pathway also plays a role during appendage regeneration in zebrafish. Increasing canonical Wnt/ß-catenin signalling, either by overactivating *wnt8* or in *axin1* heterozygous mutants, is sufficient to augment regeneration while inhibition of Wnt/ß-catenin signalling by overactivating the specific inhibitor Dkk1 leads to failure to form the blastema and to a block in regeneration [13]. In contrast, overexpression of non-canonical *wnt5b* inhibits fin regeneration, possibly by interfering with Wnt/ß-catenin signalling. In agreement, fin regeneration is accelerated in *wnt5b* homozygous mutants [13]. Therefore, a balance between canonical and non-canonical Wnt signalling seems to be required for successful fin regeneration. A big challenge now is to understand the interplay between these signalling pathways and to uncover the ways by which they are modulated during regeneration.

In this study, we have evaluated the robustness of the regenerative capacity of zebrafish caudal fins. We show that consecutive repeated amputations over a long period of time do not compromise blastema formation and outgrowth. This reveals an almost unlimited capacity to reconstitute a complex structure, possibly only limited by the life span of the fish. In addition, we challenged the regenerative capacity even further, by asking whether fin regeneration could occur normally after it has been repeatedly blocked with cycles of amputation and inhibition of Wnt/ß-catenin signalling. Once again we found that even in this extreme situation, the permanent block of regeneration caused by overexpression of Dkk1 can be relieved by a subsequent re-amputation, which then leads to normal regeneration.

## Results

### The caudal fin maintains its original size after consecutive repeated amputations

**Figure 1:**
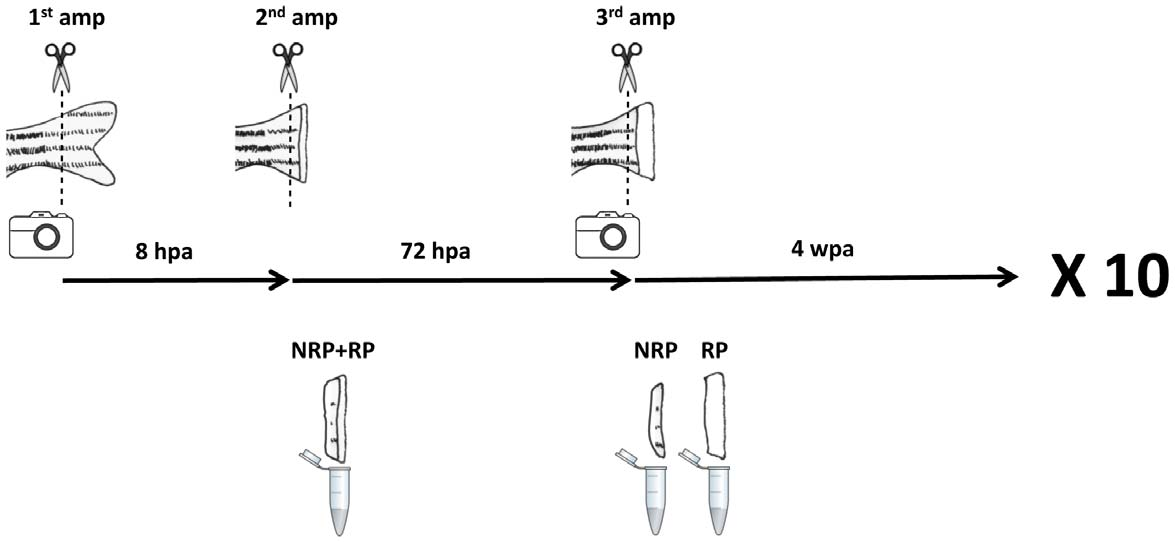
Outline of the consecutive repeated caudal fin amputations performed every month over an 11-month period. Each month, the fully regenerated caudal fin was photographed and amputated. After 8 hpa, it was subjected to a second amputation and the amputated tissue was collected. After 72 hpa, the caudal fin was photographed again, a third amputation was performed and the amputated tissues were collected. After 4 wpa, the procedure was repeated. The entire procedure was done 10 times. AMP: amputation; NRP: non-regenerate portion; RP: regenerate portion.

We designed a consecutive repeated amputation experiment to evaluate whether caudal fin regeneration is limited (Fig. 1). The caudal fin of initially 24 adult zebrafish siblings was subjected to three amputations every month. During the first 6 months the first amputation (1^st^ amp) was done one bone segment below the most proximal bony ray bifurcation. In the following months, the first amputation (1^st^ amp) was done 6 segments distally to the base of the fin. After 8 hours (8 hpa), a second amputation (2^nd^ amp) was performed to collect the regenerate portion (RP) together with stump tissue of one bone segment in length (the non-regenerate portion, NRP). After 72 hours (72 hpa), a third amputation (3^rd^ amp) was performed to collect separately the RP and the NRP to evaluate the effect of consecutive repeated amputations on regenerative outgrowth. Thereafter, we allowed the caudal fin to regenerate for 4 weeks (4 wpa) to ensure a complete regeneration. This amputation protocol was repeated 9 times spanning a period of approximately 11 months.

**Figure 2:**
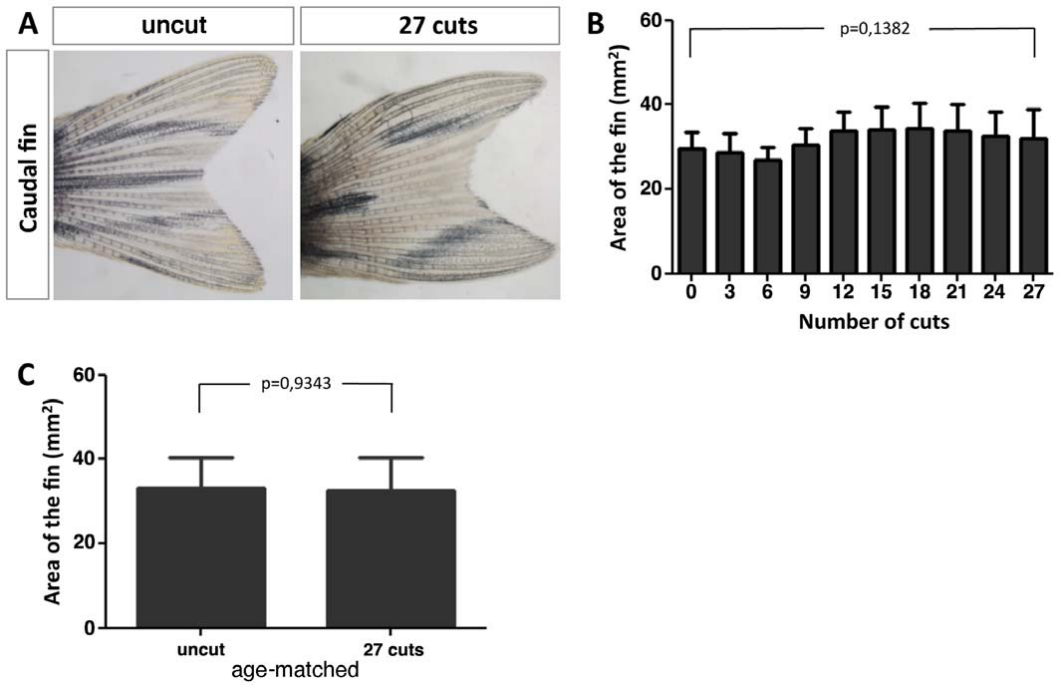
Consecutive repeated amputations maintain the original size of the fully regenerated caudal fin. (A) The same caudal fin before any amputation (0 cuts) and 4 wpa after 27 consecutive cuts. (B) Area of the 4 wpa regenerated caudal fin with increasing number of cuts. (C) Comparison of the caudal fin area of zebrafish siblings that were amputated 27 consecutive times with age matched siblings that were never amputated.

To evaluate the regenerative outgrowth state following consecutive repeated amputations, we measured every month the 4 wpa full caudal fin area of each fish. As a control, we also measured the uncut caudal fin area of each fish just before initiating the consecutive repeated amputation experiment. The area of the 4 wpa full caudal fin did not change when we compared the uncut caudal fin area (n = 24) with the one obtained after 27 cuts (n = 14) (Fig. 2A, B). To control for possible influence of fish age, we also measured the caudal fin area of zebrafish siblings (n = 10) that were never amputated but were maintained over the experimental period in the exact same conditions. Again, we found no differences in the caudal fin area of these age-matched zebrafish siblings (Fig. 2C). These results show that the regenerative outgrowth of the zebrafish caudal fin does not decline with repeated amputations.

### Blastema formation is not impaired after consecutive repeated amputations

**Figure 3:**
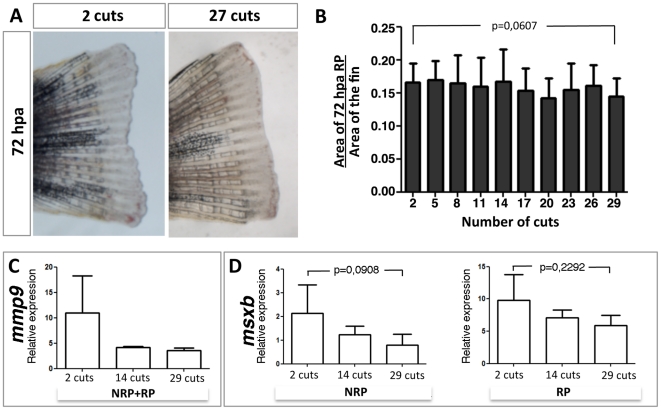
The 72 hpa regenerate size of the caudal fin is maintained with consecutive repeated amputations over an 11-month period. (A) A 72 hpa caudal fin obtained after the second consecutive amputation and after the twenty-seventh consecutive amputation. (B) Area of the 72 hpa regenerate over the area of the fully regenerated caudal fin immediately before the amputation measured with increasing number of cuts. (C) *mmp9* expression levels at 8 hpa with increasing number of cuts. (D) *msxb* expression levels at 72 hpa in both non-regenerate portions (NRP) and regenerate portions (RP) with increasing number of cuts.

We next asked whether early events after amputation, in particular wound healing and blastema formation, might be affected by repeated amputations. To this end, we measured the size of the regenerate (RP) at 72 hpa. When we correct these values for the overall individual caudal fin size by dividing the RP area by the 4 wpa full caudal fin area on each month, we found that the relative area of the 72 hpa RP did not decrease significantly even when we compared the 72 hpa RP obtained after 2 cuts (n = 24) with the one obtained after 29 cuts (n = 14) (Fig. 3A, B). To complement this data with a molecular analysis, we quantified the expression levels of the wound healing marker, *mmp9* [18] and the blastema cell marker, *msxb* [4]. Although the level of *mmp9* expression in 8 hpa NRP+RP showed a decrease after 14 cuts, this level was maintained in subsequent amputations (Fig. 3C). The levels of *msxb* also slightly decreased, even though not significantly, with increasing number of amputations (Fig. 3D). Since *msxb* is a blastema marker, it is not surprising that the levels of expression were higher in the 72 hpa RP when compared with the 72 hpa NRP (Fig. 3D). These results reveal that, even if the expression of these markers slightly decreases with repeated amputations, these changes do not result in a decline of the fin's ability to successfully accomplish wound healing and blastema formation.

### Consecutive repeated amputations affect the non-regenerated bone

**Figure 4:**
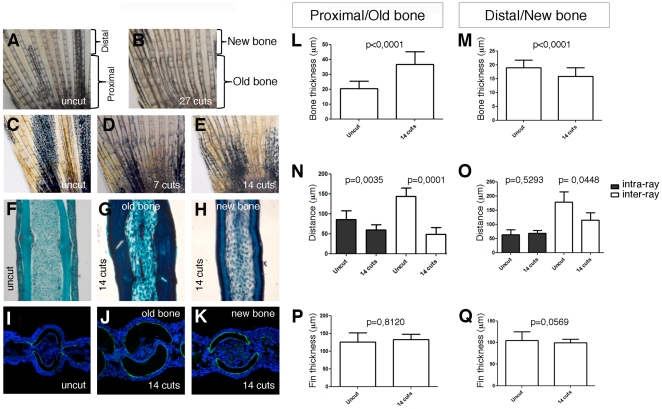
Consecutive repeated amputations affect the structure of non-regenerate bone. Picture of the dorsal lobe of an uncut caudal fin (A) and its age-matched sibling after 27 cuts (B). Picture of the dorsal lobe of an uncut caudal fin (C) and a caudal fin after 7 (D) and 14 cuts (E). Masson's trichrome staining of longitudinal sections of an uncut bony ray (F) and of an old (G) and regenerated (H) regions of a bony ray after 14 cuts. Confocal images of transverse sections of a Zns5 immunostained proximal region of an uncut caudal fin (I) and of the old (J) and new (K) tissue of a caudal fin after 14 cuts. Quantification of the bone thickness, inter- and intra-ray tissue and fin thickness in the old (L, N, P) and new (M, O, Q).

A closer look at the bony rays present in caudal fins obtained after 27 consecutive amputations revealed a clear difference between the bone segments located proximal to the amputation plane (bone that was never amputated or old bone) and bone segments located distally to the amputation plane (regenerated or new bone). Overall, old bony rays got wider and bone segment boundaries became less defined along the entire proximal-distal axis (Fig. 4B). This phenotype is not age dependent since the bony rays of uncut age-matched siblings did not change bone width and segment boundaries definition with time (Fig. 4A).

To be able to characterize and quantify the bone phenotype, we performed an independent consecutive repeated amputation experiment where two amputations were performed every other week. The first amputation of the week was always done 6 segments distally to the base of the fin and the second amputation was always done one segment below the previous one. We observed that the old bone got progressively thicker after an increased number of amputations and a clear difference between the old and the new bone was already visible after 7 cuts (Fig. 4C–E). Histological longitudinal sections of bony rays stained with Masson's trichrome expose the collagen content. This staining showed that the amount of collagen was increased in old bone (Fig. 4G) when compared with new bone regenerated after 14 cuts (Fig. 4H). Interestingly, the new bone showed a similar amount of collagen when compared to the one present in the control uncut caudal fin (compare Fig. 4H with Fig. 4F). To determine if the increase in collagen content was accompanied by an increase in the number of osteoblasts, we analysed transverse sections of caudal fins immunostained with Zns5 by confocal microscopy. A single layer of Zns5^+^ cells was found to line the bone matrix in uncut controls and in old and new bone of fins after 14 cuts (Fig. 4I–K), indicating that the number of osteoblasts lining the hemirays did not increase with repeated amputations. Quantification of the bone thickness, the space between the hemirays (intra-ray) and the space between rays (inter-ray) showed that the thickness of old bone increased significantly after 14 cuts, while the intra- and inter-ray space decreased concomitantly (Fig. 4I,J,L,N). In contrast, the regenerated new tissue presented a slight decrease in the bone thickness and a mild reduction of the inter-ray space, while the amount of intra-ray tissue is slightly increased although not significantly when compared to the uncut caudal fins (Fig. 4I,K,M,O). However the overall fin thickness, which is the sum of the bone thickness and the intra-ray space, was not affected proximally (old tissue) or distally (regenerated tissue) after 14 cuts. (Fig. 4P,Q). We conclude that repeated amputations result in abnormal remodelling of the bone and mesenchymal tissue proximal to the amputation plane.

### Regenerative capacity is not affected after repeated inhibition of caudal fin regeneration following Wnt/ß-catenin signalling perturbation

When Wnt/ß-catenin signalling is inhibited immediately after fin amputation, a wound epidermis forms, but blastema formation does not occur and regeneration is completely blocked [13], [19], [20]. We analyzed whether fin regeneration could occur normally after it has been previously perturbed.

**Figure 5:**
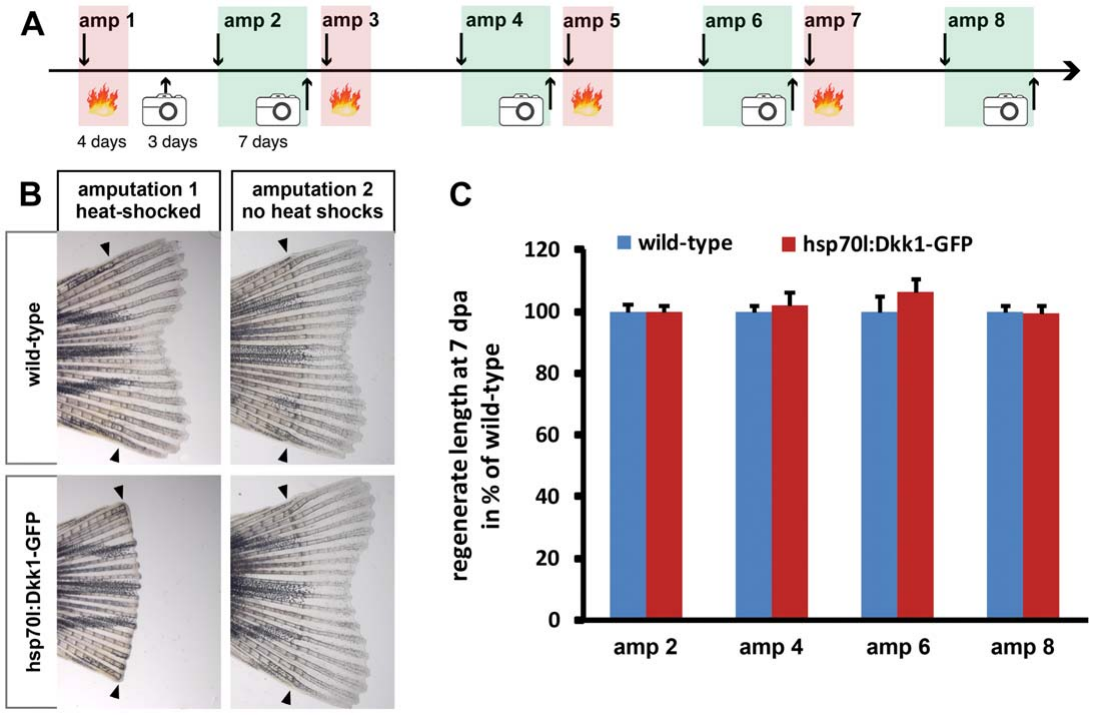
Repeated inhibition of fin regeneration by interference with Wnt/b-catenin signaling does not diminish regenerative capacity. (A) Schematic illustration of the experimental scheme. Red shaded areas indicate periods in which fish were heat-shocked twice daily, green areas indicate periods in which fish were allowed to regenerate in the absence of heat-shock. amp = amputation, phot = photo of the tail fin. (B) Wild-type and *hsp70l*:Dkk1-GFP transgenic tail fins heat-shocked until 4 dpa and photographed 7 days after amputation 1 (left column) and photographed after amputation 2 without heat-shocks (right column). Note that heat-shocked wild-type fins regenerated, while Dkk1-GFP expressing fins did not, yet both fins regenerated in the absence of heat-shocks in response to amputation 2. (C) The average regenerate length 7 days post amputation number 2, 4, 6, and 8 were normalized to the length of wild-type fish. Note that there are no significant differences in regenerate length between wild-type and *hsp70l:*Dkk1-GFP fish.

To inhibit fin regeneration, we overexpressed the Wnt pathway inhibitor Dkk1 using heat-shock inducible transgenic *hsp70l*:Dkk1-GFP fish. Overexpression of Dkk1-GFP twice daily starting shortly before fin amputation and continuing until 4 days-post-amputation (dpa) was sufficient to completely inhibit fin regeneration (amputation 1 in Fig. 5B, [13]. When fish were relieved from the heat-shock treatment, spontaneous regeneration did not occur. In contrast, when these fins that did not regenerate were re-amputated and fish were kept at non-inducing standard temperatures, fins completely regenerated (amputation 2 in Fig. 5B). Thus, the ability to regenerate after Wnt signalling inhibition requires a novel amputation stimulus. Importantly, this also shows that inhibition of Wnt/ß-catenin signalling does not permanently block the regenerative capacity of the zebrafish caudal fin. To test whether repeated cycles of regenerative inhibition caused by blockage of Wnt signalling can diminish the regenerative capacity, we repeated the cycle of amputation, heat-shocking, recovery and second amputation 4 times (Fig. 5A). We measured the length of the regenerate formed after every other amputation (in the absence of heat-shock) and plotted the length of the *hsp70l*:Dkk1-GFP transgenic regenerates normalized to the one of their wild-type siblings. As shown in Fig. 5C, no significant difference between the two groups could be detected. Thus, repeated blockage of blastema formation and fin regeneration by interference of Wnt/ß-catenin signalling did not diminish the regenerative capacity after a new amputation stimulus. We conclude that blastema formation and regenerative outgrowth do not depend on a biological process that is permanently disrupted or depleted by loss of Wnt/ß-catenin signalling.

## Discussion

Repeated amputation experiments are fundamental to uncover the regenerative capacity limit of lower vertebrates. Some reports reveal a progressive increase of defects in the regenerated limb with an increasing number of amputations in both larval *Bufo regularis* and adult *Notophthalmus viridescens* newts [21], [22]. In contrast, regeneration is successfully accomplished with only minor defects after 16 tail amputations in adult *Triturus carnifex* newts [23], [24]. This led the authors to propose that regeneration of the spinal cord in *Triturus carnifex* relies on differentiated cells present in the stump that dedifferentiate contributing to the regenerate. Whether the difference in capacity to repeatedly regenerate these structures completely without defects is due to differences between newt species or whether tails have a higher capacity to regenerate than limbs is unsolved.

Only very recently, the regeneration limit of the zebrafish caudal fin was investigated [25]. In this report, it was shown that the regenerative capacity of the zebrafish caudal fin does not decline when amputated up to 9 times. This conclusion was based on the amount of regenerated tissue at 7 dpa and on analysis of expression of *msxb* and *fgf20a* at 48 hpa. In our study, we extended these results by showing that repeated amputations up to 29 times over a period of 11 months do not alter regenerative capacity. However, in contrast to this recent report, we observed a slight decrease of expression levels of the wound healing marker *mmp9* and the blastema marker *msxb* with repeated cycles of regeneration (Fig. 3C,D). Nonetheless, these levels are still enough to accomplish a successful regeneration since the size of the 72 hpa regenerate and 4 wpa full caudal fin did not significantly change (Fig. 2). Altogether, these data show that wound healing, blastema formation and regenerative outgrowth are not affected when the caudal fin is challenged with repeated amputations. Interestingly, it was recently demonstrated that telomere length is not maintained upon 3 repeated amputations in fish older than 3 months [26]. In this scenario, one could speculate that consecutive amputations could lead to cell senescence. However, our results demonstrate the amazing regenerative potential of the zebrafish caudal fin even when challenged with a severe protocol of repeated amputations in older fish. Therefore, cell senescence can not be a limiting factor.

This almost unlimited capacity to regenerate that we have uncovered in our study could be due to either the presence of stem cells, dedifferentiation of mature cells or the contribution of both. In principle, each amputation could activate the pool of putative stem cells that might be present in different fin tissues, leading to the differentiation of all the missing structures. Importantly, the decision between self-renewal and the initiation of differentiation is controlled by signals provided by the tissue microenvironment, or niche, where stem cells are believed to reside. The Wnt signalling pathway plays a fundamental role in the control of maintenance and proliferation initiation of adult stem cells reservoirs in the intestine [27] and skin [28]. We made use of the heat-shock inducible transgenic *hsp70l*:Dkk1-GFP fish, to efficiently and in a time-controlled manner inhibit Wnt signalling. Inhibition of Wnt signalling twice daily shortly before fin amputation and until 4 dpa completely impaired fin regeneration. However, if the fins that did not regenerate were re-amputated and allowed to have an intact Wnt signalling by keeping them at a non-inducing temperature, fins regenerated completely (Fig. 5). This reveals that there is a time window for the initiation of regeneration that is triggered soon after each amputation and that is absolutely dependent on Wnt/ß-catenin signalling. Importantly, these experiments also indicate that blastema formation does not depend on a pool of progenitor cells that requires Wnt for its maintenance. While these data do not completely rule out a contribution of progenitor cells, it is more compatible with the alternative model of regeneration based on dedifferentiation. In fact, this model is now supported by recent findings showing that mature osteoblasts dedifferentiate to form part of the blastema and regenerate bone in the zebrafish caudal fin [10]. According to these findings, Wnt signalling could be required for dedifferentiation and/or expansion of the dedifferentiated cells to form a blastema.

In spite of this amazing capacity to regenerate, the bone proximally to the amputation plane becomes thickened with repeated cycles of amputations. Interestingly, we could not detect a clear difference in Zns5 staining, indicating that the number of osteoblasts did not change with increased amputations. Progressive bone thickening might be a consequence of inappropriate activation of osteoblasts to secrete matrix far away from the amputation plane. In fact there is strong evidence that osteoblasts enter the cell cycle following amputation [10], [29] and that differentiated cells can be induced to proliferate even far from the amputation plane [10], [30]. Thus, while some dedifferentiated osteoblasts migrate distally to form the blastema, it is unlikely that newly formed osteoblasts that far from the amputation plane would participate in blastema formation. Rather, they likely represent a source of cells replacing those moving into the blastema. It is possible that activation of proliferation also causes these cells to re-activate matrix secretion, which after repeated cycles results in bone thickening. Alternatively, the increase in bone matrix could be caused by an unbalanced ratio of bone-forming and bone-degrading cells. Due to the thickening of the bone, it seems that the inter- and intra-ray tissues became compacted and therefore reduced in size. Interestingly, the newly regenerated tissue of the fin exhibits a decreased bone thickness and inter-ray space probably because these are recently formed tissues that are still being remodelled.

A better understanding of the cellular mechanisms underlying the virtually unlimited regenerative capacity of fish appendage regeneration will be informative for efforts to improve repair, in particular of bone, in humans.

## Materials and Methods

### Ethics Statement

All experiments involving animals were approved by the Animal User and Ethical Committees at Instituto de Medicina Molecular, according with directives from Direcção Geral Veterinária (PORT 1005/92). All animal experiments at the Biotechnology Center of the TU Dresden were performed in accordance with the guidelines of the state of Saxony and have been approved by the Regierungspräsidium Dresden, permit number 24D-9168.11-1/2008-1.

### Zebrafish lines, maintenance and surgery

48 AB WT zebrafish were purchased from ZIRC. The repeated amputations protocol was initiated when fish were 1 year of age. 24 experimental animals were maintained at 30°C in separate tanks (one individual per tank) during the time of the experiment (approximately 11 months). 24 control uncut animals were kept together in a large tank, at the same temperature. To perform the amputations, fish were anesthetized in 0.6 mM Tricaine and amputated using a razor blade.

### Repeated inhibition of regeneration

*hsp70l*:Dkk1-GFP^w32^ transgenic fish, carrying one copy of the transgene and their wild-type siblings were used. To induce heat-shocks, fish were kept in an automated waterbath at 28°C, and twice daily heated to 37°C within 10 minutes, followed by sustained incubation at 37°C for 1 hour, and active cooling to 28°C within 15 minutes. To ensure complete block of fin regeneration in Dkk1-GFP expressing fish, the first heat-shock was applied 6 hours prior to fin amputation. To document regenerative capacity after inhibition, fish were heat-shocked twice daily for 4 days without feeding, then allowed to recover for 1 week at 28°C with feeding, followed by re-amputation of the fin in wild-types or the non-regenerated fin stump in *hsp70l*:Dkk1-GFP^w32^ transgenic fish. For re-amputation, the fin was cut 1 bone segment proximal to the initial amputation plane. Fish were allowed to regenerate with feeding at 28°C for 1 week, after which the fin was photographed.

### Quantification of regenerate area and length and caudal fin area

The 4 wpa full caudal fin and the 72 hpa regenerate area were measured each month using Image J software (NIH). Since zebrafish are very heterogeneous regarding its size, the 72 hpa regenerate area was corrected to the size of the fin by dividing its value in each month by the 4 wpa full caudal fin area in the corresponding month. The 7 dpa regenerate length of *hsp70l:*Dkk1-GFP fish was normalized to the average regenerate length of wild-type sibling fish. For this quantification, the length of the 2^nd^, 3^rd^, 4^th^ and 5^th^ dorsal fin rays was measured from the amputation plane to the distal tip of the ray using Image J software and the average length calculated for each fish.

### Quantitative RT-PCR

8 hpa RP and NRP tissues were collected and preserved at −20°C in RNA Later solution (Ambion) during the time of the experiment. Total RNA was extracted from fin regenerates using TRIZOL (Invitrogen) according to the manufacturer's protocol. 8 regenerates were used to extract RNA for the 8 hpa time-point and 4 RP or NRP were used to extract RNA for the 72 hpa time-point. 1 µg of RNA from each sample was reverse transcribed with the Revertaid™ H minus first strand cDNA synthesis kit (Fermentas) using random hexamer primers. Primers for quantitative RT-PCR of *mmp9* were 5-CTGGGCACCTGCTCGTTG-3 and 5-ATTGGAGATGACCGCCTGC-3 and for *msxb* were 5-AGGAACAGAGCACTTGGTCAAACT-3 and 5-TGAGGTTGAGGGAGTTGGAGAAC-3. Quantitative PCR was performed using Corbet Rotorgene 6000 and the SYBR Green labelling system. *mmp9* and *msxb* levels were normalized to the housekeeping gene *ef1a* (primers 5-ACGCCCTCCTGGCTTTCACCC-3 and 5-TGGGACGAAGGCAACACTGGC-3). Quantification of the relative expression was performed using the 2^−ΔCT^ method and normalized against the relative expression obtained for the uncut caudal fin. Data were analyzed using Student's t test.

### Tissue sectioning and histology

Fins were embedded in gelatin and sectioned at 12 µm using a cryostat. For the Masson's trichrome staining, gelatin was washed in PBS at 37°C for approximately 30 minutes and sections were stained with Weigert's hematoxilin for 10 minutes, washed in warm running tap water for 5 minutes and rinsed in distilled water. After this washing, sections were stained with Biebrich scarlet-acid fuchsin for 5–10 minutes. The excess of this solution was removed by rinsing with distilled water and the unspecific staining was cleared with phosphomolybdic acid 1% for 10 minutes. Collagen was stained with light green at 2% for 1 minute. Finally, sections were dehydrated in ethanol 95% 30 seconds, ethanol 100% 30 seconds, cleared in xylol for 5–10 minutes and slides were mounted in Entellan.

### Immunohistochemistry

The fins were fixed in a solution with 80% MeOH/20% DMSO (Sigma) and were rehydrated in a MeOH/PBS series, permeabilized with acetone at −20°C for 20 minutes, followed by two washes in PBS. An additional permeabilization was done with PBST 0.5% solution (PBS with 0.5% Triton X-100) during 30 minutes. Followed by several washes with PBS, fins were blocked in PBS with 10% Fetal Bovine Serum (FBS) and incubated with 1∶250 primary antibody Zns5 (ZIRC 011604) overnight at 4°C. Fins were washed several times in PBS and the incubation with the secondary antibody and DAPI (D9564 Sigma) was done overnight at 4°C. Immunostained caudal fins were post-fixed for 20 minutes in 4% PFA (paraformaldehyde), washed in PBS and passed through a 30% sucrose/PBS solution for cryoprotection. Transverse sections of 12 µm of immunostained fins of 2 uncut controls and 2 caudal fins subjected to 14 amputations were obtained by cryosectioning and analysed by confocal microscopy. In each of the controls and experimental fins the following measurements were performed using Image J software: proximal and distal bone thickness of dorsal and ventral hemi-rays of 5–9 bony rays was measured; the amount of 3 inter-ray tissues at a proximal and distal level was quantified by measuring the distance between two bony rays; the proximal and distal intra-ray tissue was quantified by measuring the length between two hemi-rays in 5–9 bony rays. Data were analyzed using Student's t-test.

## References

[1] Iovine M (2007). Conserved mechanisms regulate outgrowth in zebrafish fins.. Nature Chem Biol.

[2] Poss KD, Keating MT, Nechiporuk A (2003). Tales of regeneration in zebrafish.. Dev Dyn.

[3] Hall BK (2005). Bones and cartilage: developmental and evolutionary skeletal biology..

[4] Nechiporuk A, Keating MT (2002). A proliferation gradient between proximal and msxb-expressing distal blastema directs zebrafish fin regeneration.. Development.

[5] Handberg-Thorsager M, Fernandez E, Salo E (2008). Stem cells and regeneration in planarians.. Front Biosci.

[6] Morrison JI, Lööf S, He P, Simon AJ (2006). Salamander limb regeneration involves the activation of a multipotent skeletal muscle satellite cell population.. Cell Biol.

[7] Brockes JP, Kumar A (2002). Plasticity and reprogramming of differentiated cells in amphibian regeneration.. Nat Rev Mol Cell Biol.

[8] Echeverri K, Clarke JD, Tanaka EM (2001). In vivo imaging indicates muscle fiber dedifferentiation is a major contributor to the regenerating tail blastema.. Dev Biol.

[9] Echeverri K, Tanaka EM (2002). Mechanisms of muscle dedifferentiation during regeneration.. Semin Cell Dev Biol.

[10] Knopf F, Hammond C, Chekuru A, Kurth T, Hans S (2011). Bone regenerates via dedifferentiation of osteoblasts in the zebrafish fin.. Dev Cell.

[11] Tu S, Johnson SL (2011). Fate restriction in the growing and regenerating zebrafish fin.. Dev Cell.

[12] Kragl M, Knapp D, Nacu E, Khattak S, Maden M (2009). Cells keep a memory of their tissue origin during axolotl limb regeneration.. Nature.

[13] Stoick-Cooper CL, Moon RT, Weidinger G (2007). Advances in signaling in vertebrate regeneration as a prelude to regenerative medicine.. Genes Dev.

[14] Quint E, Smith A, Avaron F, Laforest L, Miles J (2002). Bone patterning is altered in the regenerating zebrafish caudal fin after ectopic expression of sonic hedgehog and bmp2b or exposure to cyclopamine.. Proc Natl Acad Sci.

[15] Whitehead G, Makino S, Lien C (2005). Fgf20 is essential for initiating zebrafish fin regeneration.. Science.

[16] Poss KD, Shen J, Nechiporuk A, McMahon G, Thisse B (2000). Roles for Fgf signaling during zebrafish fin regeneration.. Dev Biol.

[17] Lee Y, Grill S, Sanchez A, Murphy-Ryan M, Poss KD (2005). Fgf signaling instructs position-dependent growth rate during zebrafish fin regeneration.. Development.

[18] Yoshinari N, Ishida T, Kudo A, Kawakami A (2009). Gene expression and functional analysis of zebrafish larval fin fold regeneration.. Dev Biol.

[19] Kawakami Y, Rodriguez Esteban C, Raya M, Kawakami H, Marti M (2006). Wnt/ß-catenin signaling regulates vertebrate limb regeneration.. Genes Dev.

[20] Huang SM, Mishina YM, Liu S, Cheung A, Stegmeier F (2009). Tankyrase inhibition stabilizes axin and antagonizes Wnt signalling.. Nature.

[21] Dearlove GE, Dresden MH (1976). Regenerative abnormalities in Notophthalmus viridiscens.. J Exp Zool.

[22] Abdel-Karim AE, Michael MI (1993). Regenerative abnormalities in hind limbs of Bufo regularis induced by repeated amputations in early larval stages.. Qatar Univ Sci J.

[23] Margotta V, Filoni S, Merante A, Chimenti A (2002). Analysis of morphogenetic potential of caudal fin spinal cord in Triturus carnifex (Urodele Amphibians) subjected to repeated tail amputations.. Int J Anat Embryol.

[24] Margotta V (2004). Morphogenetic potentiality of the caudal spinal cord in adult newts (Triturus carnifex) subjected to repeated tail amputations: evaluation after the 16th amputation.. Rend Fis Acc Lincei.

[25] Shao J, Chen D, Ye Q, Cui J, Li Y (2011). Tissue regeneration after injury in adult zebrafish: The regenerative potential of the caudal fin.. Dev Dyn.

[26] Anchelin M, Murcia M, Alcaraz-Pérez F, García-Navarro E, Cayuela M (2011). Behaviour of telomere and telomerase during aging and regeneration in zebrafish.. Plos One.

[27] Korinek V, Barker N, Moerer P, van Donselaar E, Huls G (1998). Depletion of epithelial stem-cell compartments in the small intestine of mice lacking Tcf-4.. Nat Genet.

[28] Blanpain C, Fuchs E (2006). Epidermal stem cells of the skin.. Annu Rev Cell Dev Biol.

[29] Johnson SL, Bennett P (1999). Growth control in the ontogenetic and regenerating zebrafish fin.. Methods Cell Biol.

[30] Santos-Ruiz L, Santamaría JA, Ruiz-Sánchez J, Becerra J (2002). Cell proliferation during blastema formation in the regenerating teleost fin.. Dev Dyn.

